# Risk factors for Hyponatremia in COVID-19 hospitalised patients

**DOI:** 10.12669/pjms.39.1.5466

**Published:** 2023

**Authors:** Muhammad Anees, Muhammad Raza, Omair Farooq, Asim Mumtaz

**Affiliations:** 1 Prof. Dr. Muhammad Anees, FCPS. Consultant Nephrologist, Farooq Hospital Westwood Branch, Lahore, Pakistan; 2 Dr. Muhammad Raza, MBBS, Farooq Hospital Westwood Branch, Lahore, Pakistan; 3 Dr. Omair Farooq, MRCP, Farooq Hospital Westwood Branch, Lahore, Pakistan; 4 Prof. Dr. Asim Mumtaz, M.Phil. Chemical Pathology, Farooq Hospital Westwood Branch, Lahore, Pakistan

**Keywords:** COVID-19, Hyponatremia, Eunatremia, Hypoxemia, AKI, DM

## Abstract

**Objectives:**

To determine the risk factors for Hyponatremia in Coronavirus disease 2019 (COVID-19) patients.

**Methods:**

Medical records of all patients admitted in COVID-19 Isolation Intensive Care Unit of Farooq Hospital Westwood Branch, Lahore from 1^st^ July to 30^th^ September, 2020 were retrospectively reviewed. Patients with confirmed diagnosis of COVID-19 by Real Time Polymerase Chain Reaction (RT-PCR) and having Hyponatremia (serum Sodium (s/Na^+^ <135mEq/L) were included, patients with Eunatremia (s/Na^+^ within 135 - 145mEq/L) were taken as control while subjects with Hypernatremia (s/Na^+^ >145mEq/L) at admission, incomplete medical records and pregnant females were excluded from the study. Demographic, clinical and laboratory data at time of admission in hospital was extracted.

**Results:**

Of 182 included patients, 79.1% (n=144) were male 40.7% (n=74) had Diabetes Mellitus (DM) and 44.5% (n=81) were hypertensive. Forty seven percent (n=86) patients had Hyponatremia while 52.7% (n=96) were eunatremic. Forty nine percent (n=90) patients had Acute Kidney Injury (AKI) and 4.9% (n=9) patients died. Risk factors for Hyponatremia were age >60 years (OR=2.52, *p*=0.006); DM (OR=2.79, *p*=0.001); Hypoxemia (OR=3.74, *p*<0.001); Lymphopenia (OR=7.62, *p*<0.009); Hypoalbuminemia (OR=9.15, *p*<0.001); high serum Ferritin (OR=4.46, *p*<0.001), high Neutrophil to Lymphocyte Ratio (NLR) (OR=3.58, p<0.001) and AKI (OR=3.40, *p*<0.001).

**Conclusions:**

Hyponatremia was common in COVID-19 hospitalized patients. Increasing age, DM, Hypoxemia, Hypoalbuminemia, high serum Ferritin and AKI were the most significant risk factors for Hyponatremia. Hyponatremic patients had comparatively higher mortality than Eunatremic patients.

## INTRODUCTION

COVID-19 is a medical condition caused by Severe Acute Respiratory Syndrome Coronavirus-2 (SARS-COV-2). It was first encountered in Wuhan, China during December, 2019 and was declared as a pandemic by the World Health Organization (WHO) on March 11, 2020.^1^ In Pakistan, the first COVID-19 case was reported in Karachi on February 26, 2020 and the total number of confirmed cases has crossed 1,020,324 by July 30, 2021.^2^ COVID-19 is a multi-systemic disease that may involve lungs, kidneys, heart and brain. Renal manifestations of COVID-19 can be AKI, Proteinuria, Hematuria, Glomerulonephritis and electrolyte disturbances.^3^ Hyponatremia, Hypernatremia, Hypokalemia, Hypocalcemia and Hypochloremia are the most common electrolyte disorders found in hospitalized COVID-19 patients.^4^ Hyponatremia is the most frequently observed electrolyte abnormality in these patients with an incidence varying from 9.9% to 44%.^5^,^6^ Older adults particularly males, having comorbidities (DM and Hypertension), prolonged hospital stay and severe COVID-19 with associated AKI are more likely to have Hyponatremia.^5^

Multiple mechanisms are responsible for causing Hyponatremia in COVID-19 patients and the most common mechanism is Syndrome of Inappropriate Anti Diuretic Hormone (SIADH).^7^ Other causes of Hyponatremia in COVID-19 patients may include gastrointestinal sodium losses in form of vomiting or diarrhea, reduced dietary sodium intake, renal failure, diuretics therapy and cardiac or hypothalamic pituitary axis dysfunction.^7^ Hyponatremia is an independent predictor of morbidity and mortality in COVID-19 patients.^8^ Hyponatremia is preventable and reversible but life threatening complications may also develop.^8^

Globally, hyponatremia is found prevalent in COVID-19 patients with associated high morbidity and mortality. In Pakistan, medical literature regarding Hyponatremia in COVID-19 is very limited, so this study was conducted to determine the risk factors for Hyponatremia in hospitalized COVID-19 patients with an aim to improve their outcome.

## METHODS

After approval from the Institutional Review Board (IRB) dated 29/03/21, No FHWW-IRB/CU/03/2-21 the retrospective study was carried out at ICU of COVID-19 isolation department of Farooq Hospital, Westwood Branch, Lahore. Available medical records of all the patients hospitalized with COVID-19 between 1^st^ July and 30^th^ September, 2020 were reviewed. The patients with confirmed diagnosis of COVID-19 by RT-PCR through nasopharyngeal swab and with Hyponatremia (serum sodium (s/Na^+^ <135mEq/L) were included in the study while patients with Eunatremia (s/Na^+^ within 135 – 145mEq/L) were taken as control. The patients with Hypernatremia at admission (s/Na^+^ >145mEq/l), incomplete medical records and pregnant females were excluded from the study. Demographic and clinical data comprising of age, gender, co-morbidities (DM and HTN), travel history, family contact history, presenting symptoms of COVID-19, vital signs at presentation, arterial blood oxygen saturation (spO_2_) at admission, length of hospital stay and outcome were recorded. Laboratory data consisting of haematological indices {Haemoglobin (Hb) level, Total Leukocyte Count (TLC), Absolute Neutrophil Count (ANC), Absolute Lymphocyte count (ALC), NLR and Platelets to Lymphocytes Ratio (PLR)} and biochemical parameters {serum electrolytes as s/Na^+^, potassium (s/K^+^), chloride (s/Cl-), serum urea, creatinine (s/Cr) and albumin} were also taken. Data regarding inflammatory markers {serum ferritin and C-reactive protein (CRP)} was also extracted to establish the severity of COVID-19. Serum sodium, based upon its single laboratory value at the time of admission, was classified as Eunatremia (s/Na^+^ = 135 – 145 mEq/L) and Hyponatremia (s/Na^+^ < 135mEq/L). Hyponatremia was sub-classified further into mild (s/Na^+^ = 130 – 134 mEq/L), moderate (s/Na^+^ = 125 – 129 mEq/L) and severe (s/Na^+^ < 125 mEq/L).^9^ AKI was also defined according to the Kidney Diseases Initiative Global Outcome (KDIGO) guidelines^10^ as a change of ≥0.3 mg/dl in s/Cr from baseline during whole period of stay in the hospital. The minimum value of s/Cr during whole period of stay in hospital was taken as baseline. Risk factors for Hyponatremia in COVID-19 hospitalized patients were determined.

The data was entered and analyzed using IBM-SPSS V-23 and medcalc 20.009. The continuous variables were described as Mean ± SD and categorical variables as frequency and percentages. Clinical variables of subjects were compared using Student’s t-test. Odds ratios were also calculated and meta-analysis of odds ratios was done. Multivariate analysis was carried out using Logistic Regression (backward elimination method) to determine the significant predictors of Hyponatremia. A *p-*value ≤5% was taken for statistical significance.

## RESULTS

One hundred and eighty two patients who fulfilled the inclusion criteria were included in the study of which 79.1% (n=144) patients were males. The mean age of the patients was 51.42 ± 15.51 years and most of the patients 71.4% (n=130) had age <60 years. Hypertension and DM were the most common comorbidities seen in 44.5% (n=8) and 40.7% (n=74) patients respectively. Only 2% (n=4) patients had a history of travel and 10% (n=19) patients had a family contact history for COVID-19. Eighty nine percent patients (n=162) were symptomatic for COVID-19 while the remaining 11% (n=20) patients had no symptoms. The mean length of stay in hospital was 8.27 ± 4.20 days and 55.5% (n=101) patients had a stay longer than seven days. Of 182 studied patients, 47.3% (n=82) had Hyponatremia while remaining 52.7% (n=96) patients were eunatremic. Among hyponatremic group, mild, moderate and severe Hyponatremia was observed in 37.4% (n=68), 8.2% (n=15) and 1.6% (n=3) patients respectively. Forty nine percent (n=90) patients developed AKI of those 41.8% (n=76), 6.5% (n=12) and 1.1% (n=2) patients had stage I, 2 and 3 AKI respectively. The demographic, clinical and laboratory parameters that were established as risk factors for hyponatremia in COVID-19 patients are shown in Table-I.

**Table-I:** Demographic, clinical and laboratory param toeters as risk factors for Hyponatremia among COVID-19 patients.

Sr. No	Variables	Overall (N=182)	Hyponatremia 47.2%(N=86)	Eunatremia 52.8%(N=96)	T statistics	p value Hyponatremia vs Eunatremia
***Demographic and Clinical Parameters***						
1	Age (years)	51.42 ± 15.51	57.60 ± 11.92	45.88 ± 16.30	-5.484	**<0.001**
2	Length of hospital stay (days)	8.27 ± 4.20	7.77 ± 4.23	8.72 ± 4.13	1.531	0.120
3	Temperature (°F)	98.33 ± 0.78	98.31 ± 0.73	98.34 ± 0.83	0.314	0.754
4	Respiratory Rate (breaths/min)	19.96 ± 4.49	21.36 ± 4.70	18.70 ± 3.92	-4.162	**<0.001**
5	spO_2_ (%)	92.97 ± 5.32	91.83 ± 5.32	93.99 ± 5.15	2.786	**0.006**
***Laboratory Parameters***						
6	Hb (g/dL)	13.41 ± 1.67	13.19 ± 1.65	13.60 ± 1.67	1.639	0.103
7	TLC (X 10^3^/µL)	9.18 ± 3.80	9.73 ± 4.18	8.69 ± 3.37	-1.865	0.064
8	Platelets (X 10^3^/µL)	253.57 ± 92.12	252.76 ± 105.45	254.30 ± 78.86	0.112	0.911
9	ANC (X 10^3^/µL)	7.28 ± 6.57	8.52 ± 8.75	6.17 ± 3.35	-2.441	**0.016**
10	ALC (X 10^3^/µL)	1.88 ± 1.22	1.70 ± 1.54	2.05 ± 0.82	1.929	0.055
11	NLR	4.58 ± 3.52	5.47 ± 3.48	3.77 ± 3.38	-3.326	**0.001**
12	PLR	158.56 ± 79.39	176.92 ± 87.27	142.11 ± 67.94	-3.018	**0.003**
13	Serum Alb (g/dL)	3.82 ± 0.59	3.57 ± 0.40	4.04 ± 0.64	5.847	**<0.001**
14	s/Na^+^(mEq/L)	134.21 ± 4.04	131.02 ± 3.36	137.08 ± 1.86	15.239	**<0.001**
15	s/K^+^ (mEq/L)	4.17 ± 0.52	4.31 ± 0.58	4.05 ± 0.43	-3.512	**<0.001**
16	s/Cl^-^ (mEq/L)	104.01 ± 4.24	102.22 ± 4.31	105.62 ± 3.47	5.888	**<0.001**
17	CRP (mg/L)	65.95 ± 62.62	75.14 ± 59.94	57.72 ± 64.12	-1.886	0.061
18	Serum Ferritin (ng/ml)	551.42 ± 604.21	679.19 ± 606.15	436.96 ± 582.06	-2.749	**0.007**
19	Blood sugar level (mg/dl)	129.73±29.39	132.48±27.29	127.26±31.09	-1.197	0.233

A *p*-value <0.05 was considered as statistically significant.

**Fig.1:**
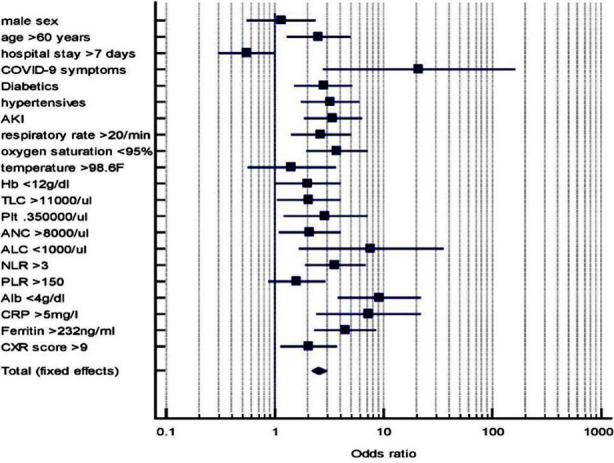
Display of ORs of Risk factors for Hyponatremia in COVID-19 Patients.

Using the univariate analysis, the statistically significant risk factors for developing hyponatremia were: increasing age, Hypertension, DM, presence of COVID-19 symptoms, tachypnea, hypoxemia, anaemia, leukocytosis, thrombocytosis, neutrophilia, lymphopenia, high NLR, hypoalbuminemia, elevated CRP, high serum ferritin and AKI as shown in Table-II along with their Odds Ratios displayed in Fig.1.

**Table-II:** OR analysis of Risk factors for Hyponatremia in COVID-19 patients.

S. No.	Variables	Unadjusted OR	95% CI	p value
1	Increasing age(>60 years)	2.52	1.29 – 4.90	**0.006**
2	Gender (Male)	1.13	0.55 – 2.33	0.727
3	Prolong hospital Stay (>7 Days)	0.54	0.30 – 0.98	**0.045**
4	COVID-19 symptoms	20.97	2.74 – 160.40	**0.003**
5	Diabetes Mellitus	2.79	1.51 – 5.14	**0.001**
6	Hypertension	3.20	1.74 – 5.89	**<0.001**
7	Fever (>98.6°F)	1.41	0.55 – 3.60	0.463
8	Tachypnoea (>20 breaths/minute)	2.63	1.39 – 4.95	**0.002**
9	Hypoxemia (<95%)	3.74	1.96 – 7.10	**<0.001**
10	Anemia (<12 g/dL)	1.98	0.99 – 3.93	**0.050**
11	Leukocytosis (>11,000/µL)	2.03	1.04 – 3.94	**0.035**
12	Thrombocytosis (>350000/µL)	2.91	1.19 – 7.09	**0.018**
13	Neutrophilia (>8000/µL)	2.09	1.09 – 3.98	**0.024**
14	Lymphopenia (<1000/µL)	7.62	1.65 – 35.11	**0.009**
15	Hypoalbuminemia(<4.0g/dL)	9.15	3.83 –21.88	**<0.001**
16	Elevated C-reactive protein(>5mg/L)	7.21	2.39 – 21.73	**<0.001**
17	High serum ferritin (>232ng/ml)	4.46	2.31 – 8.60	**<0.001**
18	High NLR (>3)	3.58	1.91 – 6.73	**<0.001**
19	High PLR (>150)	1.59	0.88 – 2.87	0.123
20	Acute Kidney Injury	3.40	1.85 – 6.26	**<0.001**
21	High CXR score (>9)	2.04	1.12 – 3.68	**0.018**

A *p*-value <0.05 was considered as statistically significant.

The multivariate analysis revealed the most significant risk factors for hyponatremia in COVID-19 were increasing age, COVID-19 symptoms, ANC, ALC, NLR and serum albumin having *p*-values of 0.006, 0.004, 0.001, 0.001, 0.023 and 0.004 respectively. Mortality was observed in nine (4.9%) patients of those six (6.97%) patients were hyponatremic while rest of the three (3.12%) had Eunatremia.

## DISCUSSION

Sodium is the most abundant electrolyte in serum and is vital for normal body functions. Sodium imbalances are common among hospitalized patients and can lead to life-threatening complications if not managed properly. Hyponatremia is the most frequently observed electrolyte disorder seen in clinical practice particularly in patients having COVID-19^11^ and is associated with increased risk of death. The manifestations of Hyponatremia can be the only first presentation of COVID-19 in certain cases.^12^ In the current study, half of the hospitalized COVID-19 patients had Hyponatremia that was almost double than that of a previous international^13^ and a local^14^ study. The high incidence of Hyponatremia in present study can be explained by following facts. At the beginning of pandemic, there was limited awareness regarding COVID-19 in society. Suspected patients having mild symptoms were reluctant from reporting to hospitals because of several misconceptions. These patients used to rely on home based therapies that failed mostly in the end.

Moreover, only some specified centers in government setups were catering COVID-19 patients but providing substandard health care services. Patient load was thus diverted to the private health sector but it was beyond financial reach of most of them. All these factors might have led to a very late presentation of most COVID-19 patients at Accident and Emergency Departments often in a critical state. In current as well as a previous^7^ study, Hyponatremia was observed more frequently in patients with severe COVID-19 that was depicted from presence of COVID-19 symptoms, Tachypnea, Hypoxemia, Leukocytosis, Neutrophilia and Lymphopenia as compared to those having milder form of disease.

Hyponatremia in COVID-19 is multifactorial.^7^ In current study, Hyponatremia might have occurred from many different mechanisms. The first and most important mechanism for Hyponatremia in the setting of COVID-19 is SIADH.^15^ In current study, raised inflammatory markers like CRP and Ferritin in severe COVID-19 probably had stimulated non osmotic ADH release thus leading to SIADH and Hyponatremia.^15^ SIADH might also be precipitated by Acute Respiratory Distress Syndrome (ARDS), respiratory failure, positive pressure ventilation and corticosteroids.^6^ A second mechanism probably involved was reduced dietary sodium intake along with gastrointestinal sodium losses (vomiting and diarrhea), although we did not collect the information regarding diarrhea and vomiting however it has been proposed to be likely in critically ill COVID-19 patients.^6^ Hyponatremia under such setting might have occurred from sodium depletion. Additionally, an increased ADH dependent free water retention induced by dehydration might have also contributed to Hyponatremia development.^6^ A probable third mechanism that might have played a role was Renin – Angiotensin – Aldosterone System (RAAS) dysfunction as seen in AKI.^16^ In the current study, half of the COVID-19 patients had AKI resulting from either a direct viral or an indirect ischemic / septic renal tubular damage. The majority of these COVID-19 patients having AKI were hyponatremic. This had also been previously observed in a study^14^ in which COVID-19 patients having AKI were found 1.2 folds more likely to be hyponatremic than those without AKI. A defective RAAS in AKI in form of an impaired renal tubular sodium reabsorption might have led to Hyponatremia.^16^ Additionally, an impaired non classic RAAS resulting from SARS-CoV-2 interaction with Angiotensin Converting Enzyme-2 (ACE-2) receptors on target cells could be the non AKI dependent mechanism of Hyponatremia in COVID-19 patients. Administration of diuretics in COVID-19 patients having volume overload might be a fourth mechanism for developing Hyponatremia through enhanced urinary sodium excretion.^16^

An increasing age is associated with an overall high morbidity and mortality. COVID-19 patients with age above 65 years suffered hyponatremia 1.9 times more than those below 50 years.^14^ In current study, nearly two thirds of COVID-19 patients older than 60 years were hyponatremic. Hyponatremia was observed more in older COVID-19 patients possibly because of their high susceptibility to COVID-19 and age related increased predisposition to Hyponatremia. Older adults are vulnerable to COVID-19 because of their immunocompromised state which is mostly secondary to some comorbid conditions. Additionally, COVID-19 in older age group usually has an atypical presentation often with superimposed complications thus making the diagnosis and management very difficult.^17^ Moreover, older people tend to have low salt intake along with medications use such as thiazide diuretics or anti-depressants known to cause hyponatremia particularly in presence of age related structural and functional changes in kidneys increasing their risk of developing AKI.^18^

COVID-19 patients with DM and HTN are respectively at 1.6^14^ and 5.5^19^ folds increased risk of developing hyponatremia. The current study showed hyponatremia in 2/3^rd^ of each diabetic and hypertensive groups. Hyponatremia in DM can be from osmosis diuresis induced hypovolemia, gastropathy associated GI losses and certain drugs like Non Steroidal Anti-inflammatory Drugs. Hyponatremia in DM can also be due to hyporeninemic hyperaldosteronism associated increased ADH release or from insulin induced Aquaporin-2 channels potentiation.^20^ Hyponatremia in HTN can be from associated chronic kidney disease, thiazides, renin secreting tumors and renal artery stenosis associated renal ischemia.^16^

COVID-19 patients are likely to be hypoalbuminemic possibly because of reduced dietary protein intake. The patients having Hypoalbuminemia are at higher risk of developing Hyponatremia than those with normal serum albumin.^5^ In current study, more than half of the hypoalbuminemic COVID-19 patients were hyponatremic. Hyponatremia in setting of a low serum albumin might have resulted from an ADH dependent free water retention. Hypoalbuminemia can induce fluid extravasation thus leading to intravascular volume depletion and an increased ADH stimulation.^21^

COVID-19 patients with hyponatremia are associated with an extended hospital stay.^22^ Hyponatremia is common in critically ill patients and in the presence of severe COVID-19 is an independent predictor of mortality.^23^ Two thirds of our patients who died had hyponatremia portraying patients with multiple complications resulting in worse outcomes.

### Limitations:

The current study was limited by its small sample size and it’s single centre based retrospective design. A multi centric prospective study with larger sample size is needed for further evaluation.

## CONCLUSIONS

Hyponatremia was common in COVID-19 hospitalized patients. Increasing age, DM, Hypoxemia, Hypoalbuminemia, high serum ferritin and AKI were the most significant risk factors for Hyponatremia. Hyponatremic patients had comparatively higher mortality than Eunatremic patients.

### Authors’ contributions:

**MA:** Main idea, conceptual frame work and final write up, drafting the work and revising it critically for important intellectual content and final approval of the version to be published.

**MR:** Data collection and data analysis.

**OF:** Literature review, helped in data analysis.

**AM:** Supervision of data collection and analysis.
